# Resilience of Gynecological and Obstetric Inpatient Care in Central Germany in Times of Repetitive Socioeconomic Shocks—An Epidemiological Study Assessing Standardized Health Services Indicators and Economic Status According to the aG-DRG Catalog

**DOI:** 10.3390/healthcare11121683

**Published:** 2023-06-07

**Authors:** Sebastian Griewing, Niklas Gremke, Michael Lingenfelder, Uwe Wagner, Corinna Keil

**Affiliations:** 1 Institute for Healthcare Management, Chair of General Business Administration, Philipps-University Marburg, Universitätsstraße 24, 35037 Marburg, Germany; 2 Department of Gynecology and Obstetrics, University Hospital Marburg, Philipps-University Marburg, Baldingerstraße, 35043 Marburg, Germany

**Keywords:** resilience, health services research, primary healthcare, gynecology, obstetrics

## Abstract

Sequential socioeconomic shocks, including the COVID-19 pandemic, economic recession, or energy and refugee crises in the face of violent conflicts, have led to the failure of healthcare systems in Europe. Against this background, the aim of this study was to evaluate the resilience of regional gynecological and obstetric inpatient care using the example of a regional core medical provider in central Germany. Base data were retrieved from Marburg University Hospital and underwent standardized calculation and descriptive statistical assessment pursuant to the aG-DRG catalog. The data illustrate a decline in the average length of patient stays and average case complexity in combination with increasing patient turnover for the six-year observation period of 2017–2022. Core profitability of the departments of gynecology and obstetrics deteriorated in the year of 2022. The results suggest weakened resilience of gynecological and obstetrics inpatient care in the setting of a regional core medical provider in central Germany and indicate how it may have failed in core economic profitability. This is consistent with predictions about the lack of resilience of health systems and the critical economic situation of German hospitals in the face of ongoing socioeconomic shocks that collaterally endanger women’s health care.

## 1. Introduction

On 23 February 2023, the Organization for Economic Cooperation and Development (OECD) published its detailed health policy report covering the lack of health systems’ resilience during the COVID-19 pandemic and following socioeconomic consequences. Since the global spread of the disease, the scientific interest in targeted investment in and restructuring of health systems’ resilience and infrastructure, to build up robustness for the upcoming crises, has skyrocketed. The capacity to absorb the effects of socioeconomic shocks on healthcare has gained new significance in times of globalization [1,2,3]. Swift global disease spread, interconnected economic crises, and the consequences of climate change subject the resilience of healthcare systems to recurring tests [2].

Hence, following the repeated socioeconomic shocks of the preceding years, worrying news hit the German hospital sector at the cusp of 2023. While still on the ropes and struggling with the COVID-19 pandemic aftermath, the national hospital questionnaire revealed rather dark conclusions about the current state of and performance predictions for German hospitals. Compared with the year of 2021, the proportion of hospitals with negative annual results was expected to rise from 43% to 59%, while positive outcomes dropped from 44% to an expected 20%. This means a reduction by half of the share of hospitals recording an annual surplus. Only 6% of German hospital managers rated their economic situation in 2022 to be rather good, while more than half of the care institutions expect their financial situation to deteriorate in 2023. Consequently, the German Hospital Association (Deutsche Krankenhausgesellschaft, DKG) fears a predicted wave of hospital insolvencies to roll in for the year of 2023. While recent data from the German Federal Statistical Office is still pending, experiences from the first pandemic year of 2020 fuel these critical predictions [4,5]. Accordingly, health expenses in the Federal Republic of Germany rose to an all-time-high of 440.6 bn € or 13.6% in terms of gross domestic product (GDP) share in 2020, financed in large part by 67.9 bn € of government transfers and grants. The government attempted to maintain the economic soundness of German hospitals via compensation payments for pandemic-related revenue shortfalls. These expenses alone accounted for 77.1% of COVID-19-related national health spending [6,7]. Omission of an interim financial stabilizer appears to render transparent the true economic state of German hospitals [8,9]. In addition, the already-failing resilience of German healthcare faced the next socioeconomic upheaval in 2022, an imminent economic recession and erratic inflation due to an energy- and refugee-crisis induced by the Russian-Ukrainian conflict.

Public health has kept a bird’s eye view on healthcare resilience, neglecting differentiated assessments for singular medical fields. Although gynecological and obstetric care contains 25,834 hospital beds, accounting for 5.3% of German inpatient care capacity, scientific consideration of previous developments on the women’s health-care situation, its resilience and financial soundness, presents as scarce and insufficient [10]. The search of most-recent literature leads back to the year of 2015, when Augurzky et al. anticipated that a decrease in inpatient cases would raise financial distress for gynecological and obstetric caretakers. Furthermore, the authors suggested accelerating care centralization and shifting cases to outpatient settings to counteract these undesired trends [11]. There has been a lack of health services and public health research covering the development of gynecological and obstetric care, evaluating economic performance, and on associated drawbacks on the care situation of women’s health against the backdrop of recent socioeconomic developments [12].

In this study, we conduct an epidemiological evaluation of gynecological and obstetric inpatient care for the catchment area of a regional core medical provider in central Germany, the Marburg University Hospital, to identify metrics for assessing associated care resilience from easily-obtainable administrative data. We investigate whether associations between spatial resilience and the performance dimension can be measured by calculating standardized health services indicators and whether these translate into the development of economic performance to identify potential setbacks to women’s health care during times of the aforementioned socioeconomic events.

## 2. Patients and Methods

### 2.1. Data Retrieval

Data extraction was performed by accessing the hospital performance controlling system QlikView^®^ (Radnor, PA, USA) and retrieving treatment data for all inpatient cases of the departments of gynecology and obstetrics of Marburg University Hospital during the six-year observation period from 2017 to 2022. Consequently, a total of n_Total_ = 36,940 patient cases were selected for assessment. Furthermore, the annual income statements of each department were collected from the internal financial-accounting department and included for further analysis.

### 2.2. Data Analysis

#### 2.2.1. Calculation of Standardized Health Services Indicators

Initially, the primary and secondary diagnoses per patient case were identified based on ICD-10 classification (International Statistical Classification of Diseases and Related Health Problems, 10th revision, World Health Organization). Thereupon, the standardized indicators of case-mix index (CMI), patient clinical complexity level (PCCL), and average length of stay (Av LoS) were calculated using Excel^®^ (Version 16.65, Microsoft Corporation, Redmond, WA, USA) for each year of observation according to the aG-DRG (German Diagnosis Related Groups) standards pursuant to §17b Section 1 sentence 4 of the German Hospital Financing Act regulations (§17b Absatz 1 Satz 4 Deutsches Krankenhausfinanzierungsgesetzes, KHG) [13]. 

##### Case-Mix Index (CMI)

CMI represents an indicator for determining average case severity and allows for conclusions with regard to the economic efficiency and resource consumption per patient. It is obtained by the division of the accumulated relative weight by the accumulated patient number per department or hospital within a specific time period. The relative weight is a defined point value determined by the aG-DRG catalog for each viable inpatient treatment and is used as a multiplier for the economic base rate (3826.61 € in 2022 for the State of Hesse, Germany) to determine the revenue per patient case (i.e., relative weight of 0.765 for a primary caesarean section without complex diagnosis, duration of pregnancy more than 33 completed weeks) [13].

$$\mathrm{CMI}=\frac{\mathrm{Case}\;\mathrm{mix}\;\left(\mathrm{CM}\right)\;\left(=\;\Sigma \;\mathrm{relative}\;\mathrm{weights}\;\mathrm{of}\;a\;\mathrm{hospital}\;\mathrm{or}\;\mathrm{department}\;\mathrm{within}\;\mathrm{time}\;\mathrm{period}\;X\right)}{\Sigma \;\mathrm{patient}\;\mathrm{number}\;\mathrm{of}\;a\;\mathrm{hospital}\;\mathrm{or}\;\mathrm{department}\;\mathrm{within}\;\mathrm{time}\;\mathrm{period}\;X}$$

##### Patient Clinical Complexity Level (PCCL)

The PCCL value is calculated based on the severity of the case-specific secondary diagnoses (complication or comorbidity-level values, CCL) in accordance with the algorithm developed as part of the CCL Refinement Project [14]. Therefore, it indicates the severity of the patient-related disease burden based on results between 0 (low PCCL) and 6 (severe PCCL). The PCCL algorithm was applied according to the current methodological recommendations of the German Institute for the Hospital Remuneration System (Institut für das Entgeldsystem in Krankenhaus, InEK) [14].

##### Average Length of Stay (AvLoS)

AvLoS was calculated in accordance with the aG-DRG catalog by cumulating the absolute days of inpatient treatment and then dividing by the corresponding number of patients for each specific observation period.

$$\mathrm{AvLoS}=\frac{\Sigma \;\mathrm{number}\;\mathrm{of}\;\mathrm{inpatient}\;\mathrm{days}\;\mathrm{of}\;\mathrm{treatment}\;\mathrm{of}\;a\;\mathrm{hospital}\;\mathrm{or}\;\mathrm{department}\;\mathrm{within}\;\mathrm{time}\;\mathrm{period}\;X}{\Sigma \;\mathrm{patient}\;\mathrm{number}\;\mathrm{of}\;a\;\mathrm{hospital}\;\mathrm{or}\;\mathrm{department}\;\mathrm{within}\;\mathrm{time}\;\mathrm{period}\;X}$$

#### 2.2.2. Calculation of Economic Core Profitability

Further analysis of the departments’ income statements was performed according to multi-level contribution-margin accounting, a well-tested and established methodology for analyzing profitability of organizational entities in cost accounting. The calculation was conducted for the separate operational units of the departments of gynecology and obstetrics according to the methodology proposed by Friedl et al. [15,16]. Further differentiation of product- and product-related fixed costs is unfeasible in a healthcare setting and has been summarized as service-related costs.

Total Revenue (Total Rev)
−Variable Costs (≙ Material)
= Contribution Margin I
−Service-Related Fixed Costs (≙ Personnel)
= Contribution Margin II
−Division-Related Fixed Costs (Dep C)
= Operating Income (Gross Profit)

As the sum of variable costs and service-related fixed costs approximates to the sum of costs of material (Material) and personnel (Personnel) in the case of a German healthcare provider, it was accumulated to the position of service costs (Serv C). Total costs (Total C) equate to the sum of Dep C and Serv C. Further internal clearings related to depreciation and amortization or the university hospital’s total operating expenses, i.e., general administrative costs, research and development or restructuring costs, and the interest expense or taxes were neglected for further assessment and calculation to limit the analysis to the specific department’s core profitability. 

In the following, the economic positions were calculated as absolute figures per department and to the denominator of the annual patient number (pp), i.e.:

$$\mathrm{Total}\;\mathrm{Rev}\;\mathrm{pp}=\frac{\;\Sigma \;\mathrm{revenue}\;\mathrm{of}\;a\;\mathrm{hospital}\;\mathrm{or}\;\mathrm{department}\;\mathrm{within}\;\mathrm{time}\;\mathrm{period}\;X}{\Sigma \;\mathrm{patient}\;\mathrm{number}\;\mathrm{of}\;a\;\mathrm{hospital}\;\mathrm{or}\;\mathrm{department}\;\mathrm{within}\;\mathrm{time}\;\mathrm{period}\;X}$$

or

$$\mathrm{Gross}\;\mathrm{Profit}\;\mathrm{pp}=\frac{\;\mathrm{Contribution}\;\mathrm{margin}\;\mathrm{III}\;\mathrm{of}\;\mathrm{hospital}\;\mathrm{or}\;\mathrm{department}\;\mathrm{in}\;\mathrm{the}\;\mathrm{year}\;\mathrm{of}\;X}{\Sigma \;\mathrm{patient}\;\mathrm{number}\;\mathrm{of}\;a\;\mathrm{hospital}\;\mathrm{or}\;\mathrm{department}\;\mathrm{in}\;\mathrm{the}\;\mathrm{year}\;\mathrm{of}\;X}$$

Descriptive statistical analysis was applied to calculate the relative change in % for prior-year (17/18, 18/19, 19/20, 20/21 and 21/22) and first-to-last year comparison (17/22) as well as pre- to post-COVID-19 time periods (17–19/20–22). Equivalently, relative changes in % of the income statement positions were calculated with the use of Excel^®^ according to the aforementioned annual comparisons.

#### 2.2.3. Visual Combination of the Normalized Development of Standardized Health Services Indicators and Economic Core Profitability

To allow for comparison of dissimilar economic and epidemiological data of the departments over the entire period under consideration, the base data of patient number (N_Obstetrics_, N_Gynecology_), AvLoS and CMI from Section 2.2.1 as well as Gross Profit from Section 2.2.2) were normalized to the first observed year, 2017. Subsequently, an aggregated graphical visualization of the corresponding development was realized by using PowerPoint (Version 16.66.1, Microsoft Corporation, PA, USA). The graphic was combined with the dates of the first registered COVID-19 case in Germany (27 January 2020), the Russian-Ukrainian conflict onset (24 February 2022), as well as time periods for aforementioned governmental compensation payments for pandemic-related revenue shortfalls in Germany (16 March 2020 to 30 September 2020, 18 November 2020 to 15 June 2021, 15 November 2021 to 18 April 2022). 

To comply with Marburg University Hospital’s compliance guidelines, data reporting of profit and loss accounts was limited to relative and normalized changes only.

## 3. Results

### 3.1. Development of Standardized Health Services Indicators

Table 1 depicts the absolute and relative development of the standardized indicators during the selected observation period. The total of N_Total_ = 36,940 patient breaks down into N_Gynecology_ = 11,342 (30.7% of N_Total_) gynecological and N_Obstetrics_ = 25,598 (69.3% of N_Total_) obstetric cases. In relative assessment, total number of obstetrics patients increases during the observation period (17/22_Obstetrics_ = 29.8%; 17–19/20–22_Obstetrics_ = 24.3%), leaving 2022 as the only year with annual prior-year decline (21/22_Obstetrics_ = −7.5%). Taking the Department of Gynecology into consideration, annual increase in patient number during the pre-COVID-19 time period (17/18_Gynecology_ = +6.3%; 18/19_Gynecology_ = +8.9%) is followed by a decline after the occurrence of COVID-19 (19/20_Gynecology_ = −6.2%; 21/22_Gynecology_ = −7.5%; 17–19/20–22_Gynecology_ = −0.7%). The assessment of standardized health services indicators indicates an overall decline in CMI and AvLoS for both medical specialties during the entire time period under investigation and pre- to post-COVID-19 comparison (AvLoS17/22_Obstetrics_ = −16.6%; AvLoS17/22_Gynecology_ = −16.3%; CMI17/22_Obstetrics_ = −19.8%; CMI17/22_Gynecology_ = −2.4%).

### 3.2. Development of Economic Core Profitability

Table 2 depicts the relative development of the multi-level contribution margin accounting positions. The data indicate a relative increase for all positions for obstetrics in las-to-first and pre- to post-COVID-19 comparisons. Taking the department of gynecology into consideration, this observation holds true for all positions except for a decline in gross profit in the pre- to post-COVID-19 comparison (GrossProfit17–19/20–22_Gynecology_ = −10.5%). Gross profit depicts the sole relative annual decline for both departments in 2022 to 2021 comparison (GrossProfit22/21_Obstetrics_ = −31.9%; GrossProfit22/21_Gynecology_ = −47.1%).

### 3.3. Combined Visualization of the Normalized Development of Standardized Health Services Indicators and Economic Core Profitability

**Figure 1:**
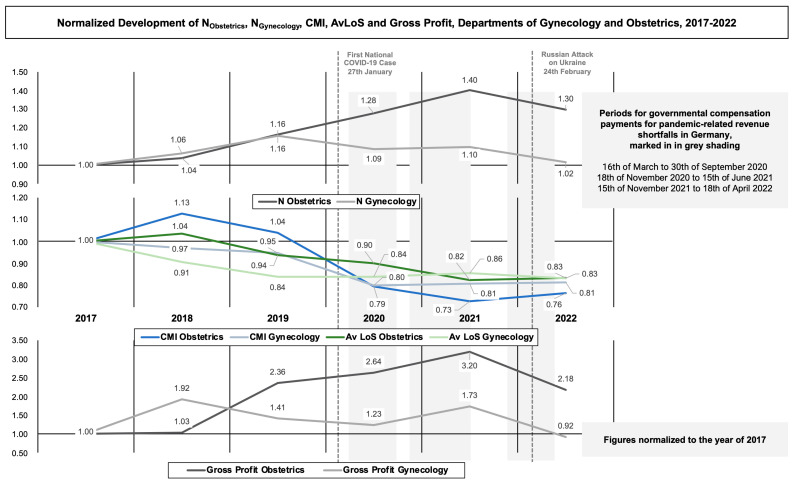
Normalized development of N_Obstetrics,_ N_Gynecology_, CMI, AvLoS and Gross Profit, Departments of Gynecology and Obstetrics, 2017–2022, normalized to the year of 2017.

Figure 1 visually links the normalized development of N_Obstetrics_, N_Gynecology_, CMI, and AvLoS to Gross Profit for comparability reasons. The normalized illustration portrays a pre-pandemic increase of N_Obstetrics_ and N_Gynecology_ (N_Obstetrics19_ = 1.16; N_Gynecology19_ = 1.16). CMI and Av LoS depict an overall decrease for both departments during the entire observation period (CMI_Obstetrics22_ = 0.76; CMI_Gynecology22_ = 0.81; AvLoS_Obstetrics22_ = 0.83; AvLoS_Gynecology22_ = 0.83). Furthermore, gross profit decreases from 2021 to 2022 in comparison with both sub-groups, leaving the last year of observation with the lowest gross profit for the department of gynecology (Gross Profit_Gynecology22_ = 0.92).

## 4. Discussion

Women’s healthcare is built on a close-knit interaction of political, environmental, and socio-economic factors [12]. It requires an accessible and interoperable network of out- and in-patient care [11]. Historically and most recently, there has been a prevailing gender inequality that goes beyond individual health choices and is influenced by structural disparities [17]. As such, although women have a longer average life expectancy in comparison to men, they spend this surplus of lifetime in poorer health [18,19]. The Gender Quality Index 2022 identifies prevailing disparities in the health dimension, setbacks in health access, and cumbersome progress of women’s health [20]. The COVID-19 pandemic has uncovered the vulnerability and the health systems’ lack of resilience to absorb socio-economic shocks [2]. Therefore, the OECD identifies the rapid growth of interdependencies at every health-system level due to ongoing globalization as a multiplicator effect. Thus, regional shocks quickly reach scales that can lead to the disruption of entire health systems [2]. There has been an upswing in health-services research efforts that search for easily-attainable and observable measures to enforce health system’s resilience by adapting theories and models of resilience to the peculiarities of healthcare [1]. Although socioeconomic shocks affect health systems on a macro level, the effects materialize in place-specific consequences on primary care, accounting for the notion of regional-care resilience [21]. So far, health services research has neglected further investigation of regional resilience of gynecological and obstetric care in light of multiple socioeconomic shocks. 

### 4.1. Main Findings

Against the background of previous neglect elaborated in research, the present study suggests weakened resilience of gynecological and obstetrics inpatient care in the setting of a regional core medical provider in central Germany, the Marburg University Hospital, by assessing standardized health services indicators according to the aG-DRG catalog for the observation period of 2017 to 2022. Furthermore, the assessment of the departments’ income statements based on multi-level contribution-margin accounting indicates failed core economic profitability following ongoing socioeconomic shocks. Beyond that, the combined visualization of the normalized development of AvLoS, CMI, patient number, and gross profit illustrates how the failed resilience of care may connect and may have caused core economic profitability failure. These main findings are discussed in detail in the following.

### 4.2. Discussion of Standardized Health Services Indicators 

CMI, AvLoS, or PCCL represent health-services indicators that are easily-attainable via standardized calculation based on data retrieved from any hospital’s information system. As such, they are widely used for controlling performance and management in DRG-based health systems, i.e., Austria, Poland, England, Ireland, France, Finland, Sweden, Spain, and many others. Nevertheless, with regard to gynecology and obstetrics, health-services research has neglected continuous monitoring and how related developments may have rendered the care situation vulnerable. The most recent health-services research literature covering the organizational state of gynecological and obstetric care in German public health leads back to 2015, when Augurzky et al. criticized the economic state of and care quality in the women’s health sector [11]. These authors requested a restructuring of gynecological and obstetric care to account for urban and rural differences in health services demand, including bundling low-complexity cases in decentralized outpatient health centers while high-complexity care should be reserved for inpatient care centers in urban areas. On this premise, Augurzky et al. identified the possibility of gynecological and obstetric care keeping pace with the increasing scarcity of skilled medical workers and of improving the economic situation as well as resilience of gynecological and obstetric care providers [11,12].

This anticipated concentration of care is reflected by an ongoing annual increase of patient numbers for the departments of gynecology and obstetrics in the case of Marburg University Hospital. Ongoing expansion of the inpatient catchment area, based on the gradual closure of peripheral care providers, has led to a fully centralized inpatient care situation, leaving the hospital as the central primary gynecological and obstetric core care provider in its region. A gradual decline in CMI shows that the upholding centralization does not push high-complexity cases to the inpatient provider but instead dilutes the average case complexity and economic efficiency. PCCL, as an additional indicator of the severity of the patient-related disease burden, presents as very low on average for gynecology and obstetrics (PCCL_Gynecology_2022 = 0.35 PCCL_Obstetrics_2022 = 0.41). This finding contradicts the recommendations of Augurzky et al. from the year of 2015 and the most recent reform proposals by the German Federal Ministry of Health (Bundesministerium für Gesundheit, BMG) from December 2022 [11,12,22]. The BMG calls for a multi-layer reorganization of the German hospital sector into differentiated levels of service provision (levels I, II, III, and III-university), putting university hospitals on top and reserving their capacity for quality care for high-complexity treatments while fulfilling their role as coordinators of the regional network of peripheral inpatient and outpatient service providers [22]. Nevertheless, the indicators point towards an opposing development during the six-year observation.

Without coherent growth of the hospital’s departments’ care capacity, the increased case load appears to be processed by lowering the average length of stay and increasing case turnover. We suppose that the dilutive effect of declining average case severity and economic efficiency, measured in combination with an increasing patient turnover, cause a setback in resilience for the regional gynecological and obstetric inpatient care in the case of Marburg University Hospital. The ongoing centralization and increasing case output contradict the desired notion of an accessible and interoperable network of out- and inpatient care but may have led to an overheating of the inpatient obstetric- and gynecological-care resilience that left the care infrastructure unable to absorb the socioeconomic shocks that started with the COVID-19 pandemic in 2020 [12]. As a result, after three years of pandemic and further crises including economic, refugee, and energy crises, the year of 2022 marks clear relative changes for both departments with an overall decrease of −7.5% in patient numbers, presumably connected to an increasing frequency of staff strikes, sick leave, as well as forced case-triaging due to care capacity decrease in the setting of Marburg University Hospital.

### 4.3. Discussion of the Economic Core Profitability 

Multi-layer contribution-margin accounting is a well-established instrument of corporate management. As a method for determining the operating result, it is an important component of every commercial controlling system and is also becoming increasingly widespread in the healthcare sector. It allows for a differentiated assessment and comparison of departmental performance in an iterative and transparent calculation of expenses and revenues towards the economic core profitability [15,16]. Thus, measured gross profit, as the key indicator of core profitability, breaks down in the annual comparison of 2022 to 2021 for gynecology (GrossProfit21/22_Gynecology_ = −47.1%) and obstetrics (GrossProfit21/22_Obstetrics_ = −31.9%). In the light of ongoing socioeconomic destabilization, this finding may only mark the beginning of an ongoing deterioration. Keeping in mind that the assessment only includes the departments’ core profitability and neglects necessary organizational investments and costs including depreciation and amortization or the hospital’s total operating expenses, and the interest expense or taxes, this observation may only show the tip of the iceberg. Overall, these findings coincide with the predictions of the German hospital questionnaire and the negative forecast of the German Hospital Association [4,5]. 

### 4.4. Discussion of the Combined Visualization of the Normalized Development of Standardized Health Services Indicators and Economic Core Profitability

Figure 1 visually combines the normalized progression of the standardized indicators with the measurement of economic core profitability. Thus, it illustrates the aforementioned combination of increasing patient turnover with decreasing average case complexity and economic efficiency by portraying patient number, CMI, and Av LoS normalized to the year of 2017. The visualization neglects PCCL for reasons of graphical clarity. It indicates the key dates of the initiation of successive socioeconomic shocks following the COVID-19 pandemic onset in 2020 and the intensification of the Russian-Ukrainian conflict in the spring of 2022. Both events entailed successive socioeconomic crises, i.e., repeated contact restrictions and lockdowns, a first-time economic recession of German price-adjusted GDP by −5.0% in 2020 after a ten-year growth period, or an ascending inflation rate of 7.9% in 2022 [23,24]. In order to financially stabilize German hospitals, the BMG granted governmental compensation payments for pandemic-related revenue shortfalls during three phases in 2020 until 2022, depicted in Figure 1. With the omission of this interim stabilizer in April 2022, the departments’ gross profit failed in 2022 in relative and normalized assessment.

This illustrated that the lack of inpatient care resilience in the setting of a medical core provider in central Germany may connect to the failure of its core economic profitability in 2022 in light of sequential socioeconomic shocks of the preceding years. Due to the high degree of inpatient care centralization, we suppose that the financial destabilization of a core provider would collaterally endanger regional women’s health care quality.

## 5. Limitations

The conducted evaluation provides considerable limitations. As such, the study design follows a single-center approach and is limited to an inpatient care setting, which sets boundaries to the transferability and generalizability of the results. Furthermore, the analysis is based on the data of an inpatient care provider in Germany and, therefore, restricts findings to health systems with DRG-based reimbursement. Based on continuous adjustment processes of the aG-DRG or ICD methodologies, biases according to miscoding cannot be completely ruled out. Moreover, further external effects in connection to fraudulent coding and misdiagnoses must be taken into account. The retrospective study design limits the evaluation to descriptive statistical assessment of regular administrative data and neglects a differentiated assessment of health utility.

Despite the single-center approach, the inpatient catchment area of Marburg University Hospital has proven to provide an adequate base for conducting regional health services research in Germany via a multitude of previous studies of the Institute for Health Care Management of Philipps-University Marburg. This leads back to the high inpatient care concentration and the balanced socioeconomic and rather rural environment of Marburg-Biedenkopf county, which compares to German standards to a sufficient degree. Furthermore, the targeted evaluation of regional resilience allows for a single-center study design.

Nevertheless, an expansion of the research design to an outpatient setting, also multi-center and multi-national analysis in subsequent studies, are desired to allow for transferability and generalizability of the findings to other health systems and urban settings. Furthermore, prospective data gathering and addition of further variables into a possible study design (i.e., mortality, patient satisfaction, time-to-diagnosis, treatment complications, etc.) would allow for statistical significance measurement of the developments and extend the analysis to the dimension of health utility assessment. 

## 6. Conclusions

Since the pandemic outbreak of COVID-19, European healthcare has been tested by sequential socioeconomic shocks including economic recession as well as related energy and refugee crises in view of the prevailing Russian-Ukrainian conflict. As a result, the failure of healthcare systems’ resilience in the face of these developments has taken on a new central role in health services research. In conjunction, the German Hospital Association fears that the economic state of its hospitals will deteriorate and collaterally endanger care quality. Being historically underrepresented, women’s health and gynecological and obstetric care have been neglected for differentiated assessment of the care resilience and consequences on economic efficiency of its care providers. By using easily-obtainable standardized health services indicators, this study investigated the weakened resilience of gynecological and obstetric inpatient care in the setting of a core medical provider in central Germany and suggests how ongoing pressure from socioeconomic shocks has led to its financial destabilization. The combination of a dilutive effect of decreasing average case complexity due to care centralization and increasing patient turnover has rendered economic efficiency critical. This pressure, following recurring socioeconomic shocks, has led to subsequent financial deterioration of the departments of gynecology and obstetrics of Marburg University Hospital in terms of gross profit in 2022. Although health services research stakeholders request a restructuring of gynecological and obstetric care for many years, i.e., to bundle low-complexity cases in decentralized care networks while high-complexity care should be reserved for inpatient maximum-care suppliers, our findings oppose these suggestions. We suggest this development would endanger care quality in women’s healthcare; therefore, we advocate for the expansion of scientific efforts to develop further and adapt innovative models of resilience to the peculiarities of gynecological and obstetric care. Financial soundness and interoperable out- and in-patient networks must be reinforced to build up regional and national resilience, to allow women’s health to absorb the effects of future socioeconomic shocks more efficiently and effectively.

## Figures and Tables

**Table 1:** Absolute and relative development of standardized health services indicators of the departments of gynecology and obstetrics, 2017–2022.

	Absolute Figures						Relative Annual Comparisons, in %						
	2017	2018	2019	2020	2021	2022	17/18	18/19	19/20	20/21	21/22	17–19/ 20–22	17/22
Overall													
N_Total_	5332	5577	6195	6476	6940	6420	4.6%	11.1%	4.5%	7.2%	−7.5%	16.0%	20.4%
Department of Obstetrics													
N_Obstetrics_	3565	3699	4150	4558	5000	4626	3.8%	12.2%	9.8%	9.7%	−7.5%	24.3%	29.8%
Av LoS	4.72	4.88	4.42	4.24	3.88	3.93	3.6%	−9.4%	−4.0%	−8.5%	1.2%	−14.0%	−16.6%
CMI	0.80	0.90	0.83	0.63	0.58	0.61	12.7%	−7.8%	−23.6%	−8.5%	5.1%	−17.5%	−19.8%
PCCL	0.25	0.26	0.31	0.37	0.36	0.41	3.0%	22.2%	18.0%	−3.1%	23.8%	−29.3%	23.8%
Department of Gynecology													
N_Gynaecology_	1767	1878	2045	1918	1940	1794	6.3%	8.9%	−6.2%	1.1%	−7.5%	−0.7%	1.5%
Av LoS	5.33	4.83	4.47	4.47	4.56	4.43	−8.9%	−7.4%	0.0%	1.9%	−2.7%	−7.8%	−16.3%
CMI	1.38	1.34	1.31	1.10	1.11	1.12	−4.4%	−2.4%	−15.6%	1.0%	0.8%	−4.4%	−2.4%
PCCL	0.50	0.50	0.44	0.39	0.43	0.35	0.7%	−12.8%	−11.9%	10.5%	−17.3%	0.7%	−12.8%

N_Total_ = Overall patient number; N_Obstetrics_ = Patient number of the department of obstetrics; N_Gynecology_ = Patient number of the department of gynecology; AvLoS = Average length of stay in days; CMI = Case mix index; PCCL = Patient clinical complexity level; Red color indicating a relative decrease, green color indicating a relative increase.

**Table 2:** Relative development of revenue, cost of revenue and gross profit of the departments of gynecology and obstetrics, 2017–2022.

	Department of Obstetrics							Department of Gynecology						
	17/18	18/19	19/20	20/21	21/22	17–19/20–22	17/22	17/18	18/19	19/20	20/21	21/22	17–19/20–22	17/22
Total Rev	2.3%	28.1%	12.5%	7.1%	−5.3%	36.4%	49.5%	9.0%	7.4%	4.6%	5.6%	−4.9%	14.9%	23.0%
*DRG Rev*	2.4%	27.0%	10.7%	7.4%	−4.6%	34.1%	47.4%	8.4%	4.9%	−3.1%	7.2%	−5.6%	5.6%	11.6%
Total C	2.2%	7.3%	12.9%	0.9%	8.6%	23.2%	35.6%	0.0%	14.5%	6.9%	1.8%	1.4%	18.7%	26.4%
Serv C	0.1%	3.3%	15.0%	2.5%	6.3%	22.1%	29.6%	6.2%	13.9%	11.5%	0.5%	4.1%	25.8%	41.2%
*Material*	−12.5%	−4.5%	21.4%	15.2%	3.2%	25.0%	20.6%	−1.5%	18.8%	7.5%	−9.1%	−9.9%	8.8%	3.1%
*Personnel*	3.0%	4.9%	13.8%	0.1%	7.0%	21.5%	31.8%	8.9%	12.3%	12.9%	3.6%	8.1%	31.5%	54.6%
Dep C	5.9%	13.9%	9.7%	−1.8%	12.3%	23.2%	35.6%	−7.1%	15.2%	0.8%	3.8%	−2.3%	9.7%	26.4%
Gross Profit	3.2%	128.7%	11.7%	21.3%	−31.9%	82.4%	117.6%	92.2%	−26.8%	−12.5%	40.6%	−47.1%	−10.5%	−8.5%
Analysis per in-patient patient (pp)														
Total Rev pp	−1.4%	14.2%	2.5%	−2.4%	2.3%	10.3%	15,2%	2.5%	−1.4%	11.5%	4.4%	2.9%	15.8%	21.1%
*DRG Rev pp*	−1.4%	13.2%	0.7%	−2.1%	3.2%	8.3%	13.6%	2.0%	−3.7%	3.4%	6.0%	2.1%	6.3%	9.9%
Total C pp	−1.5%	−4.4%	2.8%	−8.1%	17.4%	−0.8%	4.5%	−5.9%	5.1%	13.9%	0.7%	9.7%	19.7%	24.5%
Serv C pp	−3.6%	−7.9%	4.7%	−6.5%	14.9%	−1.9%	−0.1%	−0.1%	4.6%	18.9%	−0.7%	12.6%	27.1%	39.1%
*Material pp*	−15.7%	−14.9%	10.5%	5.0%	11.6%	−0.2%	−7.0%	−7.3%	9.1%	14.6%	−10.1%	−2.5%	9.5%	1.6%
*Personnel pp*	−0.7%	−6.5%	3.7%	−8.8%	15.7%	−2.3%	1.5%	2.5%	3.2%	20.4%	2.4%	16.9%	33.0%	52.3%
Dep C pp	2.0%	1.5%	−0.1%	−10.5%	21.4%	1.0%	12.5%	−12.6%	5.8%	7.5%	2.6%	5.7%	10.4%	7.7%
Gross Profit pp	−0.6%	103.9%	1.7%	10.6%	−26.4%	49.7%	67.7%	80.9%	−32.8%	−6.7%	39.0%	−42.8%	−10.3%	−9.9%

Total Rev = Total revenue; DRG Rev = revenue of inpatient treatment; Total C = Total cost of revenue; Serv C = Material + Personnel; Material = Material costs; Personnel = Cost of Personnel, Dep C = Division-Related Fixed Costs; pp = per inpatient patient; Red color indicating a relative decrease, green color indicating a relative increase; Main positions marked in gray color; Sub positions marked in italic.

## Data Availability

The datasets generated and analyzed during the current study are available from the corresponding author on reasonable request.
